# Supplementary material for: Host Genetic Background Influences BCG-Induced Antibodies Cross-Reactive to SARS-CoV-2 Spike Protein
Source: Vaccines (Basel). 2024 Feb 26;12(3):242. doi: 10.3390/vaccines12030242 (PMC10975245; doi:10.3390/vaccines12030242)
Supplement: Supplementary file 1 [file vaccines-12-00242-s001.zip › vaccines-2846613-supplementary.pdf]

Supplementary Material

Host genetic background influences BCG-induced antibodies cross-reactive to SARS-CoV-2 spike protein.

Aubrey G. Specht[1], Melanie Ginese[1], Sherry L. Kurtz[2], Karen L. Elkins[2], Harrison Specht[3], and Gillian Beamer[4*]
[1]Department of Infectious Disease and Global Health, Cummings School of Veterinary Medicine, Tufts University, North Grafton, MA, USA
[2] Center for Biologics Evaluation and Research, Food and Drug Administration, Silver Spring, MD, USA
[3] Department of Bioengineering and Barnett Institute, Northeastern University, Boston, MA, USA
[4] Texas Biomedical Research Institute, San Antonio, TX, USA

*** Corresponding author**
Gillian Beamer, VMD, PhD, DACVP
Adjunct Associate Professor
Texas Biomedical Research Institute
8715 Military Dr W, San Antonio, TX 78227

Standard Curve and Optimizing In-house ELISA:

Mouse mAb 240C binds the spike protein of the first SARS-CoV and SARS-CoV-2 [1]. We developed a standard curve using Monoclonal Anti-SARS-CoV Spike Protein Similar to 240C (BEI Resources (NR-616)) against recombinant spike protein to calculate the concentration of cross-reactive IgG in the serum of BCG-vaccinated mice. Standard curves ranged from 0-10,000 pg/mL. The lower limit of detection was 64 pg/mL calculated based on six standard curves each with triplicate wells for each known concentration (Supplemental Figure S1). Minimal variation in optical density across replicates was noted.

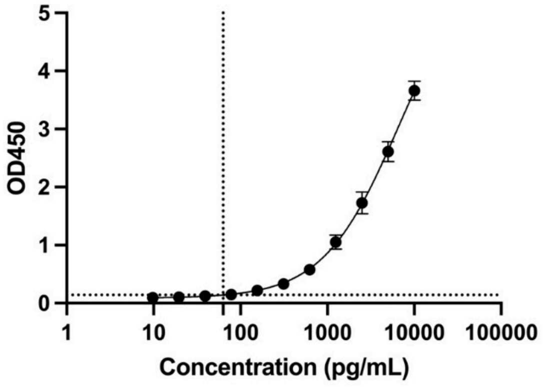

Supplemental Figure S1: Example Standard Curve using Monoclonal Anti-SARS-CoV Spike Protein Similar to 240C (mAb similar to 240C) against recombinant spike protein. Error bars show standard deviation in optical density. The limit of detection (dotted line) was 64 pg/mL.

Reference

1. Bates TA, Weinstein JB, Farley S, Leier HC, Messer WB, Tafesse FG. Cross-reactivity of SARS-CoV structural protein antibodies against SARS-CoV-2. *Cell Rep*. 2021;34(7):108737. doi:10.1016/j.celrep.2021.108737
